# Mechanical Amorphization of Chitosan with Different Molecular Weights

**DOI:** 10.3390/polym14204438

**Published:** 2022-10-20

**Authors:** Ekaterina Podgorbunskikh, Timofei Kuskov, Denis Rychkov, Oleg Lomovskii, Aleksey Bychkov

**Affiliations:** 1 Laboratory of Mechanochemistry, Institute of Solid State Chemistry and Mechanochemistry SB RAS, 18 Kutateladze Str., 630090 Novosibirsk, Russia; 2 Department of Natural Sciences, Novosibirsk State University, 2 Pirogov Str., 630090 Novosibirsk, Russia; 3 Laboratory of Physicochemical Fundamentals of Pharmaceutic Materials, Novosibirsk State University, 2 Pirogov Str., 630090 Novosibirsk, Russia; 4 Faculty of Business, Novosibirsk State Technical University, 20 Karl Marx Ave., 630073 Novosibirsk, Russia

**Keywords:** amorphization, chitosan, crystal structure, degree of crystallinity, disordering, mechanochemistry, molecular weight

## Abstract

Mechanical amorphization of three chitosan samples with high, medium, and low molecular weight was studied. It is shown that there are no significant differences between the course of amorphization process in a planetary ball mill of chitosan with different molecular weights, and the maximum degree of amorphization was achieved in 600 s of high intensity mechanical action. Specific energy consumption was 28 kJ/g, being comparable to power consumption for amorphization of cellulose determined previously (29 kJ/g) and 5–7-fold higher than that for amorphization of starch (4–6 kJ/g). Different techniques for determining the crystallinity index (*CrI*) of chitosan (analysis of the X-ray diffraction (XRD) data, the peak height method, the amorphous standard method, peak deconvolution, and full-profile Rietveld analysis) were compared. The peak height method is characterized by a broader working range but provides deviated *CrI* values. The peak deconvolution method (with the amorphous Voigt function) makes it possible to calculate the crystallinity index of chitosan with greater accuracy, but the analysis becomes more difficult with samples subjected to mechanical processing. In order to refine the structure and calculation of *CrI* by the Rietveld method, an attempt to optimize the structure file by the density functional theory (DFT) method was performed. The averaged profile of amorphous chitosan approximated by an eighth-order Fourier model improved the correctness of the description of the amorphous contribution for XRD data processing. The proposed equation may be used as a universal standard model of amorphous chitosan to determine the crystallinity index both for the amorphous standard method and for peak deconvolution of XRD patterns for arbitrary chitosan samples.

## 1. Introduction

Chitosan is a β-1,4-linked linear polymer of glucosamine extracted from crustacean shells, insects, and fungi by chitin deacetylation with concentrated alkalis (most frequently) or treatment with deacetylases (very rarely because of the high cost of enzymes) [1,2,3]. Chitosan-based materials are used for food packaging [4,5], encapsulation of food supplements [6,7], inhibition of formation of acrylamide and 5-hydroxymethylfurfural during thermal food processing [8], drug delivery [9], as a component of hemostatic agents and wound dressings [10,11], and as a material for suture-free surgery [12,13]. When used directly as a component of functional foods and therapeutic and preventive nutrition, chitosan can bind fatty and bile acids, phospholipids, and gliadin due to its ability to form micelles, thus making it possible to treat celiac disease, reduce cholesterol levels, and treat arthrosis and osteoporosis [8,14,15,16].

The properties of chitosan can be significantly altered by subjecting it to physicochemical modification such as mechanochemical disordering and amorphization [17,18,19], plasma treatment [20,21], and copolymerization [22,23]. Thus, mechanical amorphization of crystallites increases reactivity during heterogeneous processes, which are used in food, pharmaceutical, and bioengineering industries [24,25,26].

Mechanical amorphization is considered to be a highly energy-intensive method for biopolymer pretreatment; energy is inevitably spent on heating the system, grinding the material, amorphization of the crystal structure, and the course of mechanochemical reactions. Studies on energy consumption allow not only to evaluate [27,28] and improve the energy efficiency of existing technologies, but also to develop new technologies for processing amorphous-crystalline polymers [29].

However, despite the natural abundance of chitin and the fact that chitosan has been studied well and is easy to produce, its wide application is largely restrained by lack of fundamental knowledge about the relationship “composition–structure–property”. Due to numerous variations in the conditions of alkaline deacetylation of chitin, chitosan procured from different manufacturers varies in composition and structure (differing in such parameters as molecular weight, polydispersity, degree of acetylation, and degree of crystallinity). Therefore, properties of chitosan-based products are rather unstable and there can be limitations in some specific applications, especially in biomedicine [30]. Poorly characterized commercial chitosan samples are often used in the food industry; even information about the content of impurities (proteins, pigments, and inorganic salts) is usually missing for them.

Whereas spectroscopic, viscometric, and chromatographic analysis methods have been developed and implemented for determining molecular properties of chitosan (molecular weight, polydispersity, and degree of acetylation) [31,32,33], monitoring such supramolecular properties as properties of the crystal structure (identifying the polymophic modification, degrees of crystallinity or amorphization) requires using diffraction and calorimetric methods (X-ray diffraction, neutron, synchrotron diffraction analysis, and differential scanning calorimetry) [34,35,36,37,38,39,40,41,42,43]. X-ray powder diffraction (XRD) followed by analysis of XRD patterns is used most frequently to study the crystal structure of chitosan because the equipment is relatively accessible.

**Figure 1:**
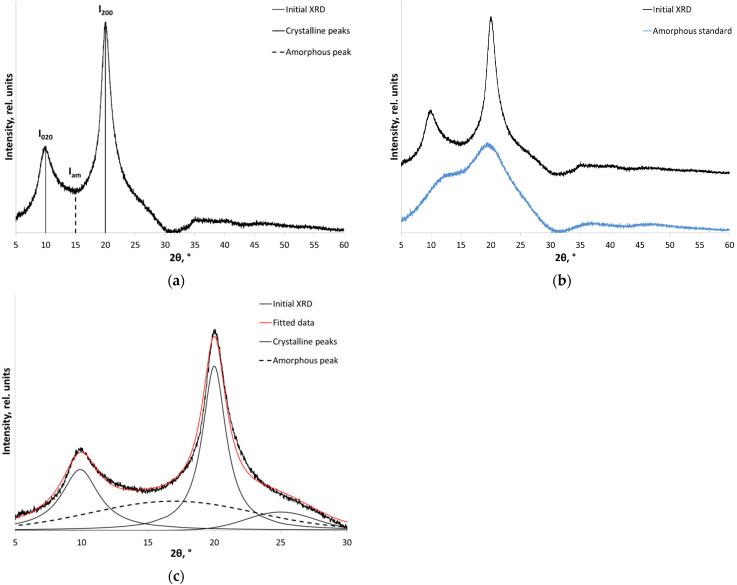
An example of fitting the XRD pattern of chitosan: using (**a**) the peak height method as proposed by Focher; (**b**) the amorphous standard method; and (**c**) the peak deconvolution method. The curves for (**b**) are shifted for clarity as indicated.

The crystallinity index (*CrI*) is most frequently determined using the peak height method that is commonly employed in cellulose chemistry. This method was first adapted to describe chitosan by Henryk Struszczyk in 1987 [44], and received recognition after the study by Bonaventura Focher [45] had been published in 1990. The method is based on the formula showing the relationship between the intensity of a crystalline reflection and intensity of the minimum peak at 2θ = 12–16°, which conditionally describes the diffuse halo peak (*I_am_*) (Figure 1a). Intensity of reflection (200) at 19–20° (Equation (1)) is determined for the most frequently used hydrated form of chitosan, while intensity of either reflection (110) at 15–16° or reflection (020) at 21–22° (Equation (2)) is determined for the anhydrous form of chitosan [32].

*CrI* = (*I*_200_ − *I_am_*)/*I*_200_, (1)

*CrI* = (*I*_110_ − *I_am_*)/*I*_110_ or *CrI* = (*I*_020_ − *I_am_*)/*I*_020_. (2)

The applicability of this method decreases substantially when the sample is a mixture of the anhydrous and hydrated polymorphs [46]. The method based on the ratio between the area of crystalline peaks and the total area of an XRD pattern (Equations (3)–(5)) has been proposed for determining the crystallinity index of the samples containing a mixture of polymorphic modifications more accurately [42,43,46].

*CrI* = *S_crystalline_*/*S_total_*, (3)

*CrI* = *S_crystalline_*/(*S_crystalline_* + *S_am_*), (4)

*CrI* = (*S_total_* − *S_am_*)/*S_total_*, (5)

where *S_crystalline_* is the area of all the crystalline peaks; *S_am_* is the area of the amorphous halo; and *S_total_* is the area of all the crystalline and amorphous peaks in the XRD pattern.

In order to directly determine *S_am_*, an XRD pattern of a deliberately amorphous sample (Figure 1b) is obtained either mechanically (long-term amorphization in a ball mill) or chemically (dissolution of chitosan in hydrochloric acid and freeze drying, followed by neutralization in an atmosphere of ammonia) [46]. Most frequently, however, the labor-intensive process of preparing and studying the amorphous standard is replaced by mathematical peak deconvolution (Figure 1c): the XRD profile is deconvoluted into peaks (deconvolution into three or four peaks is performed for the most accurate description) using the Lorentzian, Gaussian, Voigt, or pseudo-Voigt approximations [47,48,49]. The crystallinity index is calculated as the ratio of the sum of areas of crystalline peaks to the sum of areas of crystalline peaks and the amorphous components (Equation (4)). Properly determining the amorphous halo peak is the main methodological difficulty of this approach [47].

The full-profile Rietveld method is the most labor-intensive but thorough method for describing the crystal structure and determining the crystallinity index [50,51,52,53,54,55]. This method allows one to determine the structural parameters of a compound (or structures if the sample contains more than a single phase), derive scattering regions related to the crystalline and amorphous areas, and avoid measurement errors as it is possible to accurately describe the diffuse background of X-ray radiation caused by environmental exposure, apparatus bias, Compton scattering, thermal excitation of atoms and molecules, as well as structural distortions and defects. The challenge of using this method is that data about the crystal structure of the compound (unit cell parameters, the space group, and coordinates of atoms), as well as the accurate position of the reflection responsible for the amorphous phase, need to be known in advance. The crystallinity index is calculated from the ratio between the integral intensities of crystalline and amorphous scattering (Equation (6)) [56].

*CrI* = ∫*I_cr_* dθ/∫*I_total_* dθ = *F_cr_*/(*F_cr_* + *F_am_*), (6)

where *I_total_* is the total intensity of the corrected XRD pattern after the parasitic scattering background is subtracted; *I_cr_* is the intensity of crystalline scattering; *F_cr_* is the area of crystalline scattering; and *F_am_* is the area of amorphous scattering.

Therefore, the purpose of this work was to evaluate the applicability of methods for fitting the X-ray diffraction data to investigate the mechanical amorphization of three chitosan samples with different molecular weights, as well as determine specific power consumption required for complete disorder of the crystal structure.

## 2. Materials and Methods

### 2.1. Materials

Chitosans of different molecular weights were used in this study: low molecular weight (50–190 kDa, Sigma Aldrich, Product # 448869), medium molecular weight (190–310 kDa, Sigma Aldrich, Product # 448877), and high molecular weight (310–375 kDa, Sigma Aldrich, Product # 419419).

### 2.2. Scanning Electron Microscopy (SEM)

**Figure 2:**
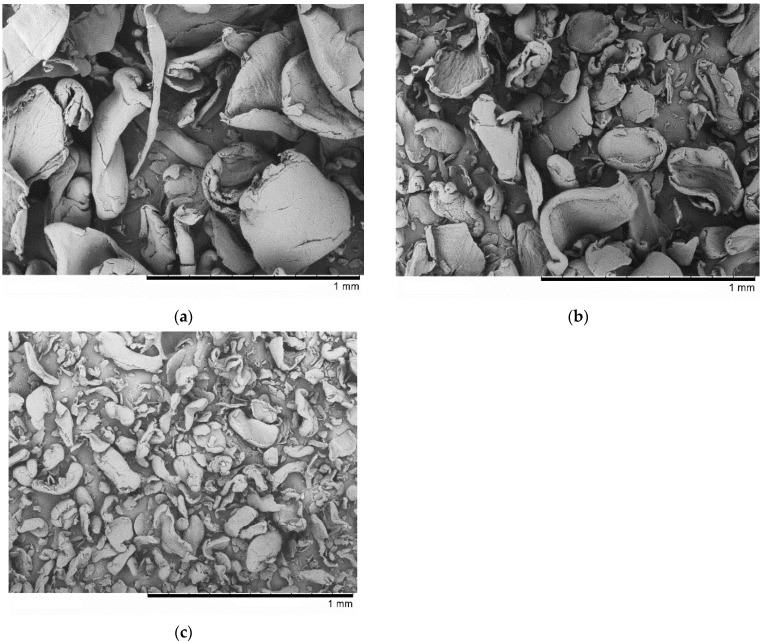
The SEM morphology of chitosan: (**a**) high MW, (**b**) medium MW, and (**c**) low MW.

The particle morphologies of chitosans of different molecular weights were studied by scanning electron microscopy (SEM) on a TM-1000 microscope (Hitachi, Tokyo, Japan) at an accelerating voltage of 15 kV. A gold coating was deposited onto the sample surface to remove the accumulated charge (ion current, 30 mA; spray time, 30 s). The SEM micrographs of chitosans of different molecular weights are shown in Figure 2.

### 2.3. Mechanical Treatment

The samples were subjected to mechanical treatment in an AGO-2 water-cooled planetary ball mill (grinding body acceleration, 200 m/s^2^; nominal motor power, 1.1 kW). Volume of the grinding jar was 135 mL. Steel balls (diameter, 5 mm; weight, 200 g) were used as grinding bodies. The weight of treated material was 10 g; duration of mechanical treatment was varied from 0 to 1200 s.

### 2.4. Power Consumption

Energy consumption for mechanical treatment was measured using a high speed wattmeter (Incotex Electronics Group, Moscow, Russia) connected to a DVP-SA2 industrial controller (Delta Electronics, Inc., Taipei, Taiwan) using the ModBus protocol.

### 2.5. X-ray Powder Diffraction (XRD)

The X-ray powder diffraction patterns were recorded on a D8 Advance diffractometer (Bruker, Karlsruhe, Germany) with monochromatic CuKα radiation in the Bragg–Brentano reflection geometry at a voltage of 40 kV and current of 40 mA; X-ray radiation wavelength was 1.5406 Å; step size was 0.0195°. The contribution of the instrument background was taken into account by subtracting XRD from the cell (a holder).

### 2.6. Density Functional Theory (DFT)

To perform Rietveld analysis, chitosan experimental structure file was obtained from ref. [38]. Missed hydrogen atoms were added using the Mercury 2021.3.0 software [57]. To avoid possible inconsistencies in crystal structure (e.g., disagreement in calculated and experimental d-spacing, symmetry, and atom positions, including partial (2/3) occupancies of water molecules) reported in [58], periodic DFT calculations were performed, resulting in crystal structure optimization to local energy minima. All DFT calculations were carried out using VASP 5.4.4. Refs. [59,60,61,62] employing the functional of Perdew, Bruke, and Ernzerhof (PBE) [63], a plane-wave basis set with a kinetic energy cutoff of 550 eV and the projector augmented wave atomic pseudopotentials [64,65] with D3BJ empirical dispersion correction [66]. The integrals in the reciprocal space were calculated on a Monkhorst–Pack mesh of 2 × 1 × 2 k-points [67], more tight convergence criteria for maximum change in system energy of 10^−5^ eV were applied. Crystal structure optimization was performed with fully relaxed (ISIF = 3), volume fixed (ISIF = 4), and fixed (ISIF = 2) unit cells. Taking into account that water molecule occupancy is 2/3 for three different water positions in the initial structure, the chitosan structure was also presented as a superposition of three structures with pairs of water molecules (e.g., positions 1 and 2 with occupancies 1, positions 1 and 3 with occupancies 1, and positions 2 and 3 with occupancies 1). Thus, the structure with three water molecules and three structures with two water molecules in different positions were optimized using three different procedures (devoted to possible changes in the unit cell) and used for Rietveld analysis [68] and as the initial experimental structure.

## 3. Results and Discussion

### 3.1. Mechanical Treatment of Chitosan

**Figure 3:**
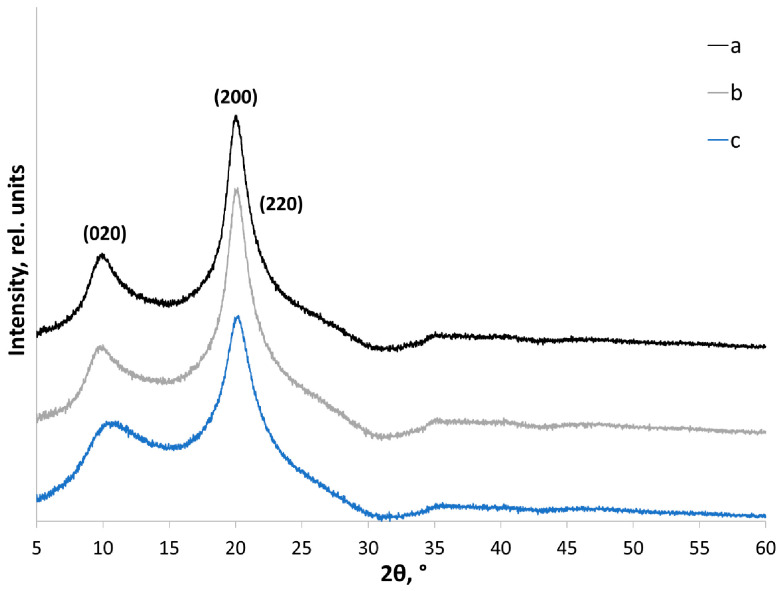
X-ray powder diffraction patterns of initial chitosan: (a) high MW; (b) medium MW; and (c) low MW. The curves are shifted for clarity as indicated.

For chitosan used in this study, reflections corresponding to crystals in the hydrated form are detected at 2θ ~ 10° (020) and 20° (200). The individual (220) reflection is not observed when recording XRD patterns under these conditions but can be seen as a shoulder on the side of the (200) reflection (Figure 3).

**Figure 4:**
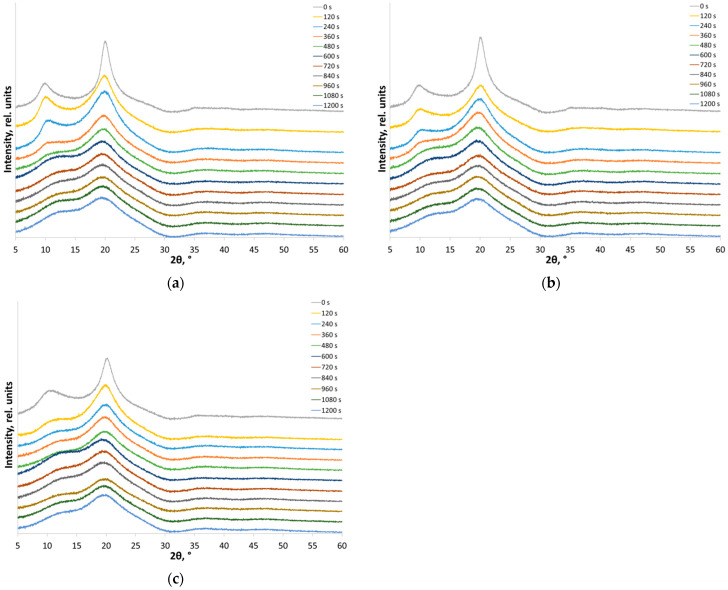
X-ray powder diffraction patterns of chitosan ((**a**) high MW, (**b**) medium MW, (**c**) low MW) subjected to mechanical treatment in a planetary ball mill for 0–1200 s. The curves are shifted for clarity as indicated.

Treatment of amorphous-crystalline polymers (cellulose, starch, chitin, and chitosan) in a planetary ball mill has proved to be a good method for producing deliberately amorphous samples [51,69]. This approach enables reproducible fabrication of samples to adequately deconvolute the integral area (*S_total_*) into the components: the crystalline areas (*S_crystalline_*) and the diffuse amorphous halo (*S_amorphous_*). In this study, the amorphous standard was prepared by mechanical treatment of chitosan in a planetary ball mill for 20 min (Figure 4).

The XRD patterns (Figure 4) show the qualitative changes in the crystal structure taking place as the amorphous-crystalline chitosan samples are exposed to intense mechanical treatment. The intensity of reflections of the crystalline phase decreased with longer treatment duration, while the line corresponding to the amorphous phase was broadened, and a diffuse amorphous halo appeared. In the XRD patterns of low MW chitosan, the (020) peak at 2θ = 10° disappeared after 2 min of treatment, while the (200) peak was shifted towards smaller angles. This shift in the position of the maximum of a crystalline peak is typical of the amorphous phase state of polymers and was previously observed for mechanically treated cellulose [68]. For chitosan samples with medium and high MW, similar changes are observed after a longer treatment (~8 min). Additional data showing mechanical amorphization are presented in the Supplementary Materials (Figure S1).

### 3.2. Fitting Methods for Chitosan X-ray Powder Diffraction Patterns

#### 3.2.1. The Focher Method, the Amorphous Standard Method, and Peak Deconvolution

Since the chitosan used in this study is represented by the hydrated polymorphic modification, Equation (1) was employed to calculate the crystallinity index using the Focher method (Figure 1a). The intensity of the minimum describing the diffuse halo (*I_am_*) was fixed at 2θ = 14°. Table 1 summarizes the crystallinity indices for the initial and mechanically treated chitosan samples with different molecular weights. The crystallinity indices of the initial high, medium, and low MW chitosan were 80, 77, and 63%, respectively. The sensitivity of the Focher method allows one to accurately assess the crystallinity of the samples up to crystallinity index of ~30–35%. Meanwhile, it has been demonstrated that under the identical conditions of mechanical treatment in a planetary ball mill, the absolute crystallinity index decreases more significantly for high and medium MW chitosan than for low MW chitosan.

**Figure 5:**
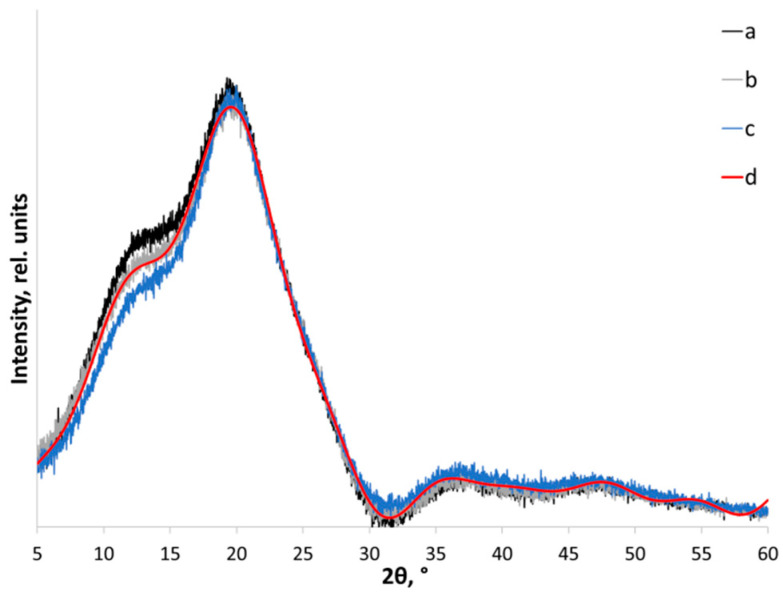
X-ray powder diffraction patterns of high (a), medium (b), low (c) MW chitosan after mechanical amorphization for 1200 s, and (d) amorphous profile approximated by the eighth-order Fourier model (“universal amorphous standard”).

The amorphous standard method usually allows one to obtain more detailed and accurate information about the crystalline nature of amorphous-crystalline material from the XRD data. To perform methodologically adequate refinement of the content of the crystalline fraction of polymers, one should use the samples as non-texturized powders, subtract the parasitic X-ray background, and perform adjustment of the XRD pattern, including subtracting the instrument background from diffraction of the cell (the holder) [56]. Proper fitting of the XRD patterns (with empty holder XRD intensities subtracted) of amorphized chitosan samples (Figure 4a–c) showed that the profile of XRD patterns did not change even after mechanical treatment for 720 s, thus proving that the amorphous state was attained for each sample (Figure 5).

The X-ray powder diffraction patterns of amorphous standards of each chitosan sample after the intensity of XRD of a cell (an empty holder) had been subtracted were described as the eighth-order Fourier series. The equation is shown below (Equation (7)), while the respective coefficients are provided in the Supplementary Materials (Table S1).

F(x) = a0 + a1*cos(x*w) + b1*sin(x*w) + a2*cos(2*x*w) + b2*sin(2*x*w) + a3*cos(3*x*w) + b3*sin(3*x*w) + a4*cos(4*x*w) + b4*sin(4*x*w) + a5*cos(5*x*w) + b5*sin(5*x*w) + a6*cos(6*x*w) + b6*sin(6*x*w) + a7*cos(7*x*w) + b7*sin(7*x*w) + a8*cos(8*x*w) + b8*sin(8*x*w), (7)

where *x* is 2θ (in degrees); the calculations of sin(n*x*w) and cos(n*x*w) are based on the input data (in radians).

The respective amorphous samples were used to calculate the crystallinity index by the amorphous standard method (Table 1). It is methodologically important to use this equation for fitting the experimental data after subtracting the background intensity and normalizing the intensity minima in the profiles of the analyzed sample and the amorphous standard at 2θ = 14–15° and ~31–32°.

It can be assumed that the similarity of the profiles of amorphous samples of chitosan with different molecular weights and the close Fourier series coefficients allow one to obtain the averaged profile of an amorphous sample, which can be further used as a “universal amorphous standard” for calculating the crystallinity index of an arbitrary chitosan sample. After subtracting the profile of the cell (the empty holder), the resulting averaged profile of an amorphous chitosan sample was fitted using the eighth-order Fourier series; the coefficients are summarized in Table 2. It was demonstrated that the equation with coefficients for the averaged amorphous standard (Table 2) can be used for routine studies or when it is impossible to obtain the amorphous standard for the analyzed substance, but provides somewhat underestimated *CrI* values for high MW chitosan and overestimated *CrI* values for low MW chitosan.

Processing the XRD patterns by deconvolution is a complex problem involving deconvolution into crystalline peaks and properly describing the profile of contribution of the amorphous phase. Deconvolution of XRD patterns using the Voigt function (Figure 1c), which is a result of the Gaussian and Lorentzian functions, allows one to describe crystalline peaks with a high accuracy. Meanwhile, it is impossible to describe the contribution of the amorphous phase using this function, which inevitably causes an error in determining the crystallinity index. Thus, the crystallinity index of high MW chitosan determined using the deconvolution method with coefficient of determination *R^2^* = 0.9878 was 67%. *CrI* of the medium and low MW chitosan was 65% with *R^2^* = 0.9929 and 63% with *R^2^* = 0.9976, respectively. To improve the accuracy of describing the amorphous profile, one can use function (7) with parameters specified in Table 2 and Table S1. However, analysis of the XRD data by the deconvolution method is impeded as one proceeds to the mechanically treated samples.

Hence, the Focher method allows one to estimate the crystallinity index of chitosan without any additional processing of XRD patterns, up to the crystallinity indices reduced to ~30%. Long-term mechanical treatment of chitosan with different molecular weights in a ball mill makes it possible to average the profile of XRD patterns of amorphous samples; in terms of shapes and positions of the peaks, the profiles correspond to the behavior of amorphized polymers (e.g., chemically amorphized chitosan [46] and cellulose [51]). Therefore, the resulting averaged amorphous samples can be used as standards to routinely determine the crystallinity index.

#### 3.2.2. Full-Profile Rietveld Analysis

**Figure 6:**
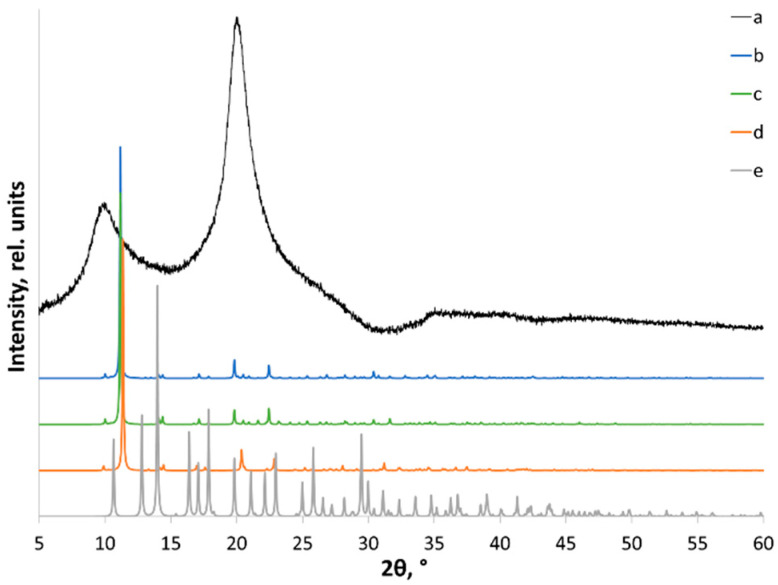
X-ray powder diffraction patterns of (a) initial XRD; (b) calculated from the structure reported in [38]; and (c) DFT optimized with fixed unit cell parameters; (d) DFT optimized with fixed unit cell volume; and (e) fully optimized DFT.

Although the Rietveld method and its various modifications have been successfully used for characterizing *CrI* of cellulose [53], its applicability for common crystalline-amorphous samples and robustness are debatable [51]. Moreover, the refinement procedure requires structure files of high quality, which is still an unsolved task for chitosan. This issue might be solved by performing various DFT optimizations of an experimental structure file. The calculated X-ray powder diffraction pattern from a structure file [38], as well as the DFT-optimized one (in multiple ways, see Materials and Methods), differ significantly from the experimental patterns and cannot be recommended for Rietveld analysis (Figure 6, Figure S2 and Table S2 of Supplementary Materials).

The lack of high quality structural data and problems related to distortion of unit cell parameters upon DFT optimization of the structure make it impossible to use the full-profile Rietveld analysis method to estimate *CrI* of chitosan.

### 3.3. Power Consumption for Amorphization of Chitosan

**Figure 7:**
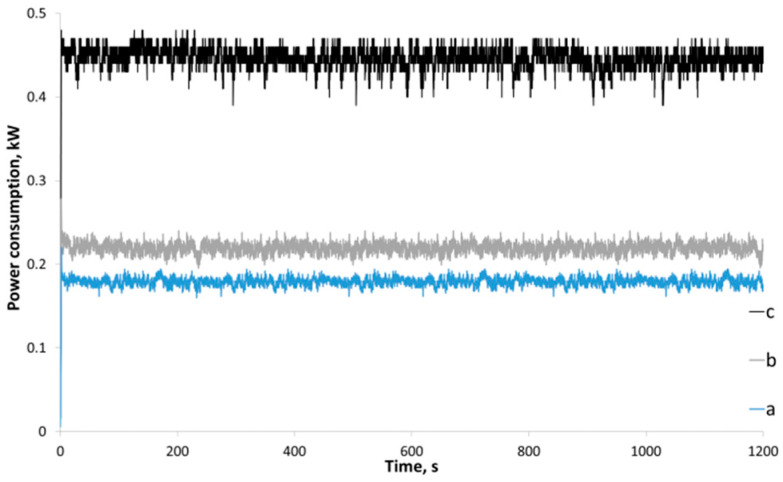
Current power consumption of the planetary ball mill: (a) the planetary gear without jars; (b) the planetary gear with empty jars; (c) jars containing grinding bodies and medium MW chitosan.

The consumed power of a ball mill and total energy consumption for amorphization of chitosan with different molecular weights were measured using a high speed wattmeter. The recorded power consumption curves (Figure 6) show that the ball mill attains the steady-state mode appreciably quickly, which is important when studying amorphization in a short treatment duration. Power consumption differs twofold for the ball mill with empty jars and the ball mill with jars loaded with 200 g of steel grinding bodies + 10 g of chitosan, but power consumption is independent of the type of chitosan being treated (Figure 7c shows the current power consumption during mechanical amorphization of medium MW chitosan, but the current power consumption of all the chitosan samples is provided in Figure S3 in Supplementary Materials).

**Figure 8:**
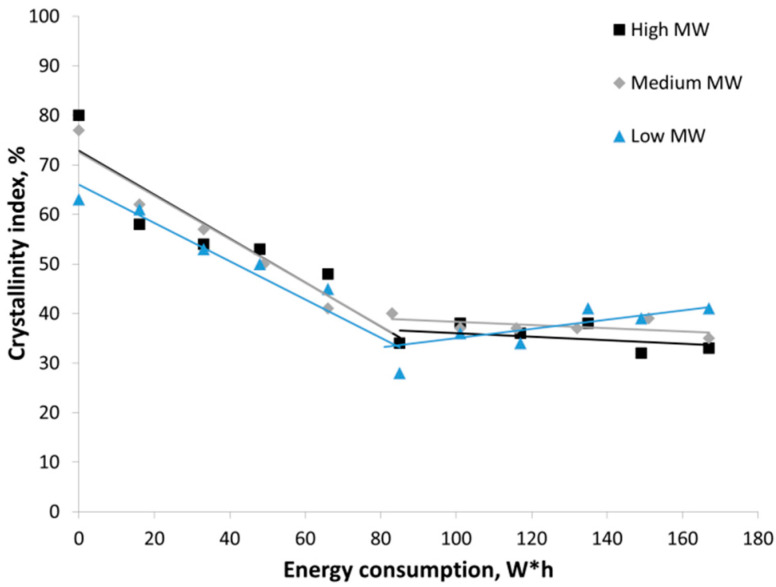
Changes in the crystallinity index of high, medium, and low MW chitosan as a function of power consumption during mechanical treatment.

A more vivid presentation of the amorphization process can be made by plotting changes in the crystallinity index (Figure 8) as a function of energy consumption during mechanical treatment. The relative amorphization degree (*AD*) was calculated as the ratio of changes in the crystallinity index to the initial crystallinity index using Equation (8):

*AD* = (*CrI*_0_ − *CrI_t_*)/*CrI*_0_, (8)

where *AD* is the amorphization degree; *CrI*_0_ is the initial crystallinity index; and *CrI_t_* is the crystallinity index after mechanical treatment.

One can see that there are no considerable differences between the amorphization of chitosan with high, medium, and low MW. The maximum amorphization degree (*AD* of ~50%) is attained within ~600 s; the specific power consumption is ~28 kJ/g. It is five- to sevenfold higher than power consumption for amorphization of polymorphic modifications of starch (4–6 kJ/g) [28] and is comparable to power consumption for amorphization of cellulose [29] whose supramolecular structure is very similar to that of chitosan.

## 4. Conclusions

This study addressed the amorphization of three chitosan samples with different molecular weights during mechanical treatment in a planetary ball mill. The methods for determining the crystallinity index by analyzing the XRD data were compared. The peak height method is characterized by a larger operating range but causes deviation in *CrI* values for the samples consisting of a mixture of polymorphic modifications. The amorphous standard method and deconvolution using the Voigt function allow one to calculate the crystallinity index of chitosan more accurately. Taking into account multiple inconsistencies in the suggested structure file of chitosan, it is not recommended to use it (as well as periodic DFT optimized structures) for Rietveld analysis.

Long-term mechanical treatment of chitosan with different molecular weight yields X-ray diffraction patterns with weakly differing profiles, so one can obtain an averaged amorphous chitosan and use it as the standard to routinely determine the crystallinity index of arbitrary chitosan samples.

The similarity of the crystalline structure of the analyzed chitosan samples suggests that specific power consumption values required to attain the limit of amorphization do not differ significantly and are equal to 28 kJ/g, being comparable to the previously measured power consumption for amorphization of cellulose (29 kJ/g) and five- to sevenfold higher than power consumption for amorphization of polymorphic modifications of starch (4–6 kJ/g).

## Figures and Tables

**Table 1:** The crystallinity indices of the initial and mechanically amorphized chitosan with different molecular weights.

Duration of Treatment in a Planetary Ball Mill, s	CrI, %					
	High MW Chitosan		Medium MW Chitosan		Low MW Chitosan	
	Focher Method	Amorphous Standard Method	Focher Method	Amorphous Standard Method	Focher Method	Amorphous Standard Method
0	80 ± 2	44 ± 2	77 ± 2	37 ± 3	63 ± 2	23 ± 3
120	58 ± 2	20 ± 3	62 ± 2	28 ± 3	61 ± 2	13 ± 3
240	54 ± 2	18 ± 4	57 ± 2	19 ± 3	53 ± 3	12 ± 3
360	53 ± 2	15 ± 2	50 ± 3	10 ± 2	50 ± 3	AM
480	48 ± 2	13 ± 4	41 ± 4	AM	45 ± 3	AM
600	34 ± 3	AM *	40 ± 5	AM	28 ± 6	AM
720	38 ± 2	AM	37 ± 3	AM	36 ± 4	AM
840	36 ± 3	AM	37 ± 3	AM	34 ± 3	AM
960	38 ± 2	AM	37 ± 4	AM	41 ± 3	AM
1080	32 ± 3	AM	39 ± 4	AM	39 ± 4	AM
1200	33 ± 4	AM	35 ± 5	AM	41 ± 5	AM

* AM—the completely amorphous material; the crystallinity indices are below the sensitivity of the method.

**Table 2:** The coefficients of the eighth-order Fourier model for fitting the XRD data of the averaged amorphous chitosan.

	Value	Standard Error	t-Value
a0	2057.08	1.85	1115.21
a1	−914.44	7.46	−122.50
a2	−1172.64	5.41	−216.62
a3	543.35	2.91	186.80
a4	−365.26	1.83	−199.88
a5	114.82	2.07	56.93
a6	151.68	1.91	79.60
a7	−146.86	1.90	−77.34
a8	−40.94	4.77	−8.58
b1	2316.04	1.87	1236.22
b2	−1023.79	7.43	−137.71
b3	18.32	4.80	3.82
b4	19.11	7.04	2.71
b5	−151.88	1.76	−86.34
b6	47.14	1.95	24.16
b7	104.88	4.17	25.18
b8	−136.97	2.11	−65.00
w	0.1092	1.41 × 10^−4^	776.5742

*R*^2^ = 0.9992; reduced chi-sqr (*χ*^2^) = 3674.5296.

## Data Availability

Not applicable.

## Supplementary Materials

The following supporting information can be downloaded at: https://www.mdpi.com/article/10.3390/polym14204438/s1, Figure S1: DSC curves of chitosan ((a) high MW, (b) medium MW, (c) low MW) subjected to mechanical treatment in a planetary ball mill for 0, 720, and 1200 s; Figure S2: Predicted XRD patterns for crystal structures obtained using different initial geometries and optimization options. The initial structure was obtained from ref. [1] and used as an input file for different DFT optimization procedures. Water position shows which water positions are occupied (123 means all three positions are occupied, while 12 means that position 3 is vacant). Full_Opt is for full unit cell optimization (ISIF = 3), Vol_Fix is for volume (but not cell parameters) of the fixed unit cell (ISIF = 4), Cell_Fix is for no cell optimization (ISIF = 2). Atom positions were free to optimize in all calculations via the VASP 5.4.4 package; Figure S3: Current power consumption of the planetary ball mill: (a) the planetary gear without jars, (b) the planetary gear with empty jars; (c) jars containing grinding bodies and low MW chitosan; (d) jars containing grinding bodies and medium MW chitosan; (e) jars containing grinding bodies and high MW chitosan. Curves “d” and “e” are shifted upward by 0.05 and 0.1 kW, respectively, for clarity as indicated; Table S1: The coefficients of the eighth-order Fourier model for fitting the XRD of amorphous chitosans; Table S2: The parameters of the crystal structure obtained using different initial geometries and optimization options. Water position shows which water positions are occupied (123 means that all three positions are occupied, while 12 means that position 3 is vacant). Full_Opt is for full unit cell optimization, Vol_Fix is for volume (but not cell parameters) of the fixed unit cell, Cell_Fix is for no cell optimization. Atom positions are free to optimize in all calculations. Italics are used for parameters that were fixed in a certain procedure.
