# Supplementary material for: Mechanical Amorphization of Chitosan with Different Molecular Weights
Source: Polymers (Basel). 2022 Oct 20;14(20):4438. doi: 10.3390/polym14204438 (PMC9606905; doi:10.3390/polym14204438)
Supplement: Supplementary file 1 [file polymers-14-04438-s001.zip › polymers-1951118-supplementary.pdf]

polymers

Mechanical Amorphization of Chitosan with Different Molecular Weights

Ekaterina Podgorbunskikh [1,*], Timofei Kuskov [1,2], Denis Rychkov [1,3], Oleg Lomovskii [1] and Aleksey Bychkov [1,4,*]

1 Laboratory of Mechanochemistry, Institute of Solid State Chemistry and Mechanochemistry SB RAS, 18 Kutateladze Str., 630090 Novosibirsk, Russia
2 Department of Natural Sciences, Novosibirsk State University, 2 Pirogov Str., 630090 Novosibirsk, Russia
3 Laboratory of Physicochemical Fundamentals of Pharmaceutic Materials, Novosibirsk State University, 2 Pirogov Str., 630090 Novosibirsk, Russia
4 Faculty of Business, Novosibirsk State Technical University, 20 Karl Marx Ave., 630073, Novosibirsk, Russia
* Correspondence: podgorbunskikh@solid.nsc.ru (E.P.); bychkov.a.l@gmail.com (A.B.)

Table S1. The coefficients of eighth-order Fourier model for fitting the XRD data of amorphous chitosans.

	High MW chitosan		Medium MW chitosan		Low MW chitosan		Coefficients obtained by averaging the XRD patterns of amorphous high, medium, and low MW chitosans (Table 2)	
	Value	Standard Error	Value	Standard Error	Value	Standard Error		
a0	2104.627	1.92309	2043.183	1.8006	2026.002	1.94075	2057.084	1.84456
a1	-900.359	7.5597	-863.168	7.71334	-988.725	7.50582	-914.435	7.46477
a2	-1265.86	5.65079	-1181.57	5.07546	-1061.3	5.9494	-1172.64	5.41334
a3	520.7729	2.70404	516.5861	2.998	589.9593	3.25257	543.3516	2.90869
a4	-343.512	1.60828	-373.187	1.95155	-379.746	2.03154	-365.258	1.8274
a5	143.6971	1.87071	105.512	1.94916	97.54484	2.37864	114.8187	2.01673
a6	159.5398	1.84367	139.7876	2.0161	155.6665	1.94993	151.6756	1.90555
a7	-151.386	1.76535	-135.79	2.03167	-154.348	1.98581	-146.855	1.89892
a8	-32.4264	4.77135	-49.8505	4.56132	-34.3203	5.28759	-40.9378	4.77242
b1	2459.837	1.70375	2350.022	1.85346	2136.736	2.2464	2316.039	1.87349
b2	-1044.94	7.61494	-966.749	7.53414	-1069.53	7.49423	-1023.79	7.4345
b3	-22.8011	4.77059	20.21194	4.63262	65.27874	5.28926	18.31606	4.79972
b4	-27.8319	6.75228	23.23158	7.19097	53.40703	7.58847	19.11425	7.04378
b5	-140.946	1.81559	-149.495	1.78987	-163.475	1.83272	-151.883	1.75917
b6	58.50624	2.05676	30.1486	1.81232	56.49073	2.21486	47.14009	1.95135
b7	100.1234	4.11561	108.5313	4.15564	100.7944	4.44548	104.8801	4.16536
b8	-137.131	1.87529	-129.399	2.39191	-144.336	2.09265	-136.969	2.10719
w	0.10961	1.40E-04	0.10881	1.41E-04	0.10956	1.50E-04	0.10926	1.41E-04

Table S2. The parameters of crystal structures obtained using different initial geometries and optimization options. Water position shows which water positions are occupied (123 means that all three positions are occupied, while 12 means that position 3 is vacant). Full_Opt is for full unit cell optimization, Vol_Fix is for volume (but not cell parameters) of the unit cell fixed, Cell_Fix is for no cell optimization. Atom positions are free to optimize in all calculations. Italics are used for parameters that were fixed at a certain procedure.

Water_positions	Form	a	b	c	alpha	beta	gamma	V
**123**	*Input_Cif*	*8.95*	*16.97*	*10.34*	*90.00*	*90.00*	*90.00*	*1570.45*
	Full_Opt	7.12	13.82	10.37	90.00	90.01	90.00	1021.33*
	Vol_Fix	8.72	17.24	10.45	90.00	90.00	90.00	*1570.45*
	Cell_Fix	*8.95*	*16.97*	*10.34*	*90.00*	*90.00*	*90.00*	*1570.45*
**12**	Input_Cif	8.95	16.97	10.34	90.00	90.00	90.00	1570.45
	Full_Opt	6.10	15.17	10.60	90.00	90.00	90.00	980.48*
	Vol_Fix	7.87	19.04	10.48	90.00	90.00	90.00	*1570.45*
	Cell_Fix	*8.95*	*16.97*	*10.34*	*90.00*	*90.00*	*90.00*	*1570.45*
**13**	Input_Cif	8.95	16.97	10.34	90.00	90.00	90.00	1570.45
	Full_Opt	7.60	15.95	10.60	90.00	90.00	90.00	1284.78*
	Vol_Fix	8.99	16.55	10.56	90.00	90.00	90.00	*1570.45*
	Cell_Fix	*8.95*	*16.97*	*10.34*	*90.00*	*90.00*	*90.00*	*1570.45*
**23**	Input_Cif	8.95	16.97	10.34	90.00	90.00	90.00	1570.45
	Full_Opt	6.33	14.95	10.46	90.00	90.00	90.00	989.97*
	Vol_Fix	9.58	15.61	10.50	90.00	90.00	90.00	*1570.45*
	Cell_Fix	*8.95*	*16.97*	*10.34*	*90.00*	*90.00*	*90.00*	*1570.45*

* Full DFT optimization leads to significant unit cell shrink in *a* direction and cannot be recommended for the chitosan system. Such significant deviation in unit cell parameters can be explained by substantial inaccuracies in the initial chitosan structure file. This is another argument for not using Rietveld refinement in this case.

Differential scanning calorimetry (DSC) analyses were carried out in argon gas atmosphere. Approximately 2 mg of native tapioca starch and starch-based films were weighed in hermetic pans in order to avoid water loss. An empty hermetic pan was used as a reference. Samples were heated from 25 to 400°C at a heating rate of 10 °C/min in the argon atmosphere.

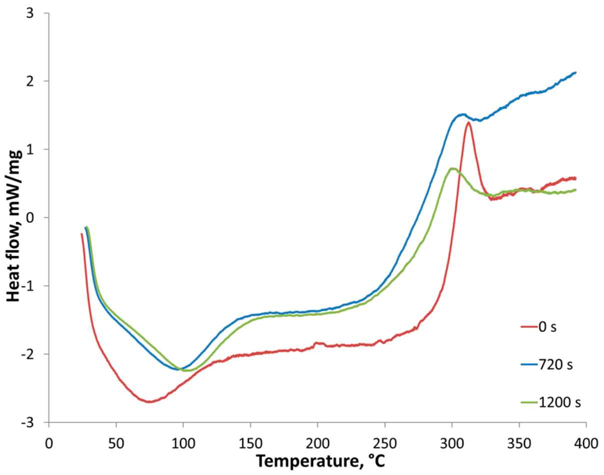

(**a**)

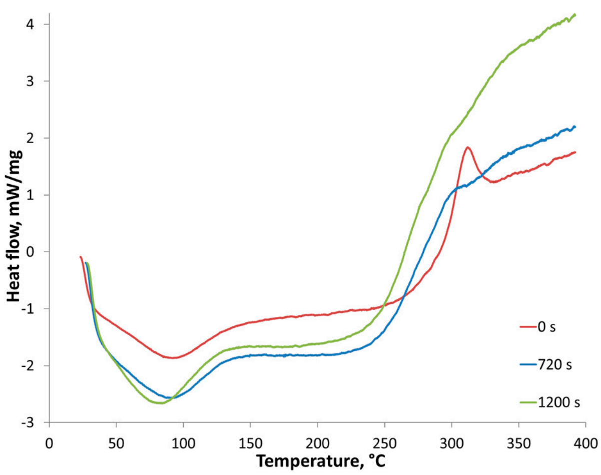

(**b**)

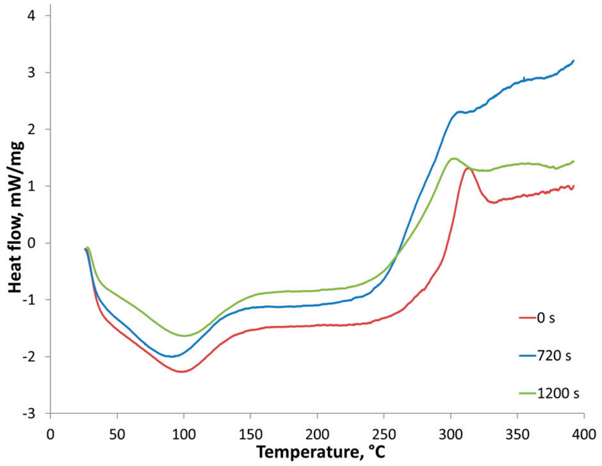

(**c**)

Figure S1. DSC curves of chitosan ((**a**) high MW, (**b**) medium MW, (**c**) low MW) subjected to mechanical treatment in a planetary ball mill for 0, 720, and 1200 s.

Differential scanning calorimetry (DSC) analyses were carried out in argon gas atmosphere. Approximately 2 mg of native tapioca starch and starch-based films were weighed in hermetic pans in order to avoid water loss. An empty hermetic pan was used as a reference. Samples were heated from 25 to 250°C at a heating rate of 10 °C/min in the argon atmosphere.

polymers

MDPI

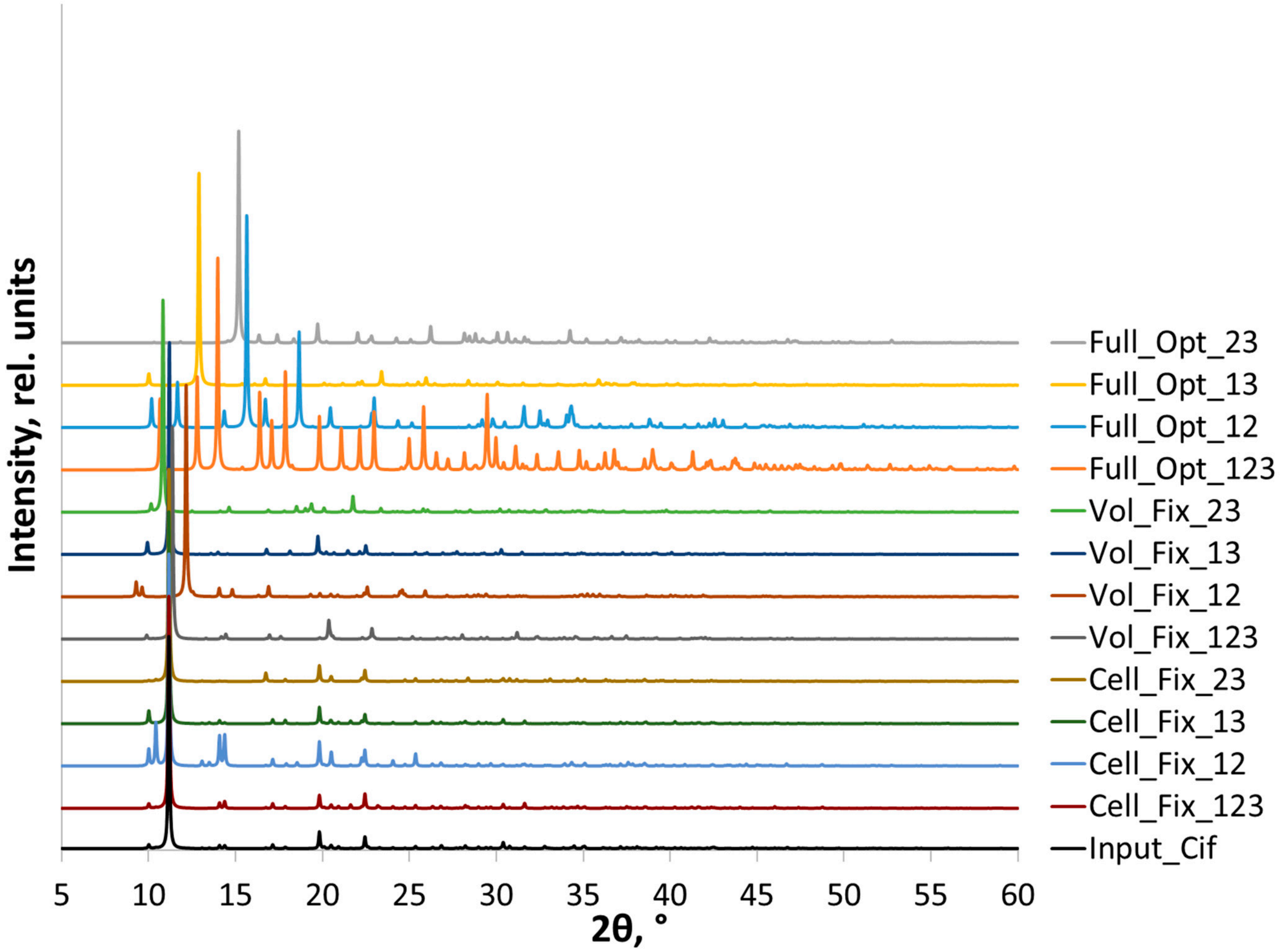

Figure S2. Predicted XRD patterns for crystal structures obtained using different initial geometries and optimization options. The initial structure was obtained from [*] and used as input file for different DFT optimization procedures. Water position shows which water positions are occupied (123 means that all three positions are occupied, while 12 means that position 3 is vacant). Full_Opt is for full unit cell optimization (ISIF=3), Vol_Fix is for volume (but not cell parameters) of the unit cell fixed (ISIF=4), Cell_Fix is for no cell optimization (ISIF=2). Atom positions were free to optimize in all calculations via the VASP 5.4.4 package.

* Okuyama, K.; Noguchi, K.; Miyazawa, T.; Yui, T.; Ogawa, K. Molecular and crystal structure of hydrated chitosan. *Macromolecules* **1997**, *30*, 5849–5855. https://doi.org/10.1021/ma970509n

polymers

MDPI

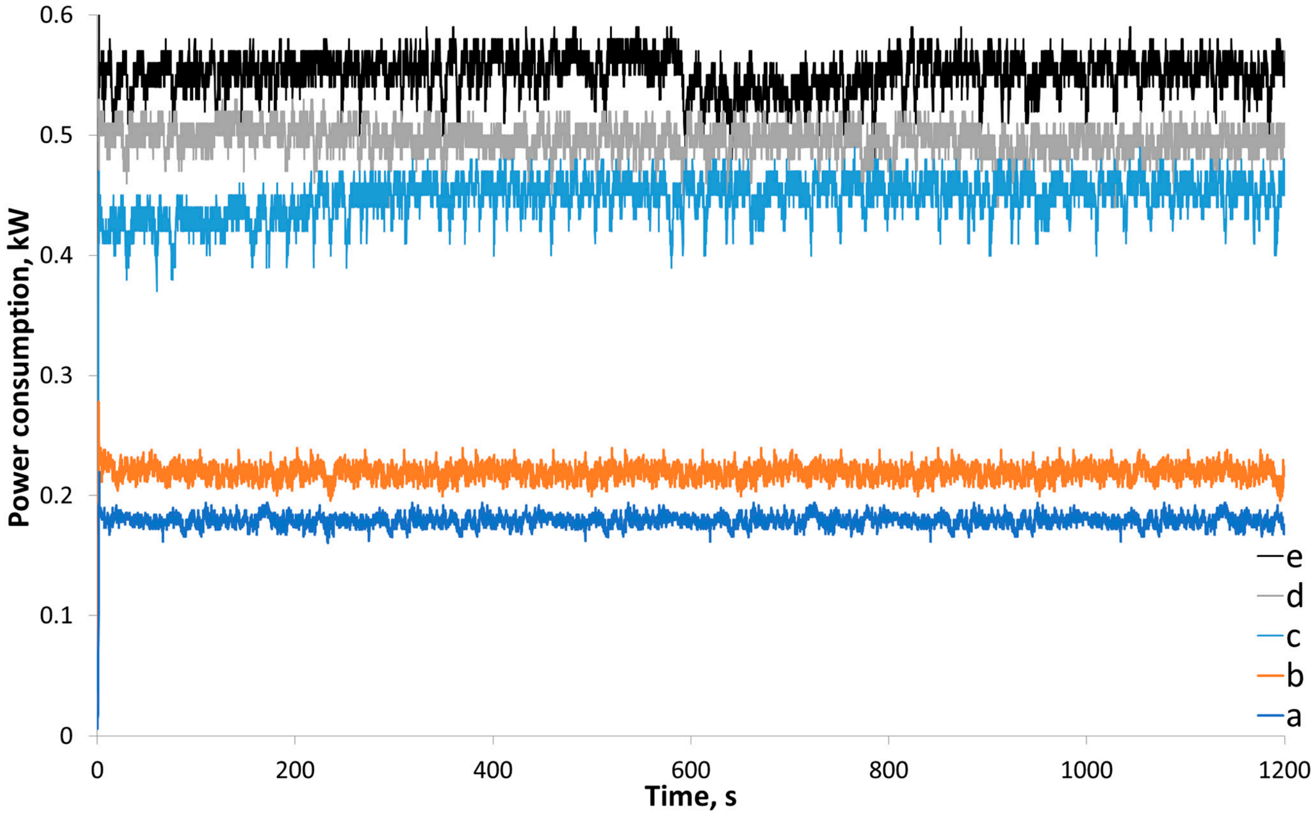

Figure S3. Current power consumption of the planetary ball mill: (**a**) the planetary gear without jars, (**b**) the planetary gear with empty jars; (**c**) jars containing grinding bodies and low MW chitosan; (**d**) jars containing grinding bodies and medium MW chitosan; (**e**) jars containing grinding bodies and high MW chitosan. Curves "**d**" and "**e**" are shifted upward by 0.05 and 0.1 kW, respectively, for clarity as indicated.
